# Ustekinumab is effective in the treatment of linear psoriasis: a case report and literature review

**DOI:** 10.3389/fimmu.2025.1553793

**Published:** 2025-04-22

**Authors:** Jinxu Qi, Qing Zhu, Mengdi Feng, Lijuan Liu, Guoqiang Zhang

**Affiliations:** ^1^ Department of Dermatology, The First Hospital of Hebei Medical University, Shijiazhuang, Hebei, China; ^2^ Subcenter of National Clinical Research Center for Skin and Immune Diseases, Shijiazhuang, Hebei, China; ^3^ Hebei Provincial Innovation Center of Dermatology and Medical Cosmetology Technology, Hebei, Shijiazhuang, China

**Keywords:** psoriasis, Blaschko line, ustekinumab, inducement, treatment

## Abstract

Linear psoriasis is a rare type of psoriasis that usually presents as scaly papules and plaques unilaterally distributed along the Blaschko line. Currently, the pathogenesis of linear psoriasis is not fully understood, and genetic mosaicism may be one of the explanations. As linear psoriasis is less responsive to treatment than psoriasis vulgaris, there is no consensus on treatment guidelines specifically for this disease, and the efficacy of ustekinumab in treating this category has not been reported. In this article, we present a case of effective treatment of linear psoriasis with ustekinumab and review the relevant literature, hoping to help clinicians better understand and treat this disease.

## Introduction

Linear psoriasis is a rare subtype of psoriasis first described by Clark in 1922. Typical clinical manifestations are scaly papules and plaques arranged continuously or intermittently, often unilaterally in a linear pattern along the Blaschko line. It can be divided into two types: type I, also known as the isolated type, is characterized by linear lesions, which are the only manifestations and not transformed from other types of psoriasis. Commonly, the patient had no history of psoriasis vulgaris. The second one—type II—also known as the superimposed type, is characterized by linear lesions that are superimposed on less severe psoriasis lesions, and after anti-psoriasis treatment, they will become visible when the psoriasis lesions subside. The patient’s condition may be more complex and does not respond well to treatment, necessitating long-term follow-up and prevention of comorbidity occurrence (1). The pathogenesis of the disease is unclear and the genetic mosaicism theory is currently accepted (2). It is treated with anti-psoriasis therapy, including topical corticosteroids, vitamin D_3_ derivatives, retinoids, and systemic drugs such as methotrexate and acitretin capsules. However, linear psoriasis responds poorly to these conventional treatments and biologics, with only a small proportion of patients achieving more than 50% improvement. Due to the limited number of cases, the response rate to biologics is rarely reported (3).

Ghoneim et al. (4) published a case report on the successful treatment of linear psoriasis with ixekizumab, bringing new hope for using novel biologics and personalized treatment for patients with linear psoriasis. However, to date, no cases of successful treatment of linear psoriasis with ustekinumab have been reported. Ustekinumab is an IL-12/IL-23 inhibitor, mainly used for the treatment of moderate to severe psoriasis and inflammatory bowel disease. As we all know, IL-23 is the core cytokine driving psoriasis, and by activating Th17 cell function, it promotes the release of pro-inflammatory factors such as IL-17A, leading to epidermal hyperplasia and inflammatory infiltration; IL-12 can promote the release of IFN-γ through the Th1 pathway, disrupting the skin barrier. Therefore, the dual inhibitory role of ustekinumab may provide a more comprehensive effect in complex or refractory cases (5, 6). It can also significantly increase body cellular mass (BCM) and phase angle (PhA), improving systemic metabolic status and promoting skin repair (7). Compared to tumor necrosis factor-α inhibitors, it is advantageous in reducing psoriasis lesions but not in joints (8), and it still improves skin symptoms even after 24 months of treatment, suggesting that its long-term modulation of the immune system may prevent disease progression (9). In this article, we report a case of a 15-year-old male adolescent who was diagnosed with linear psoriasis and treated with ustekinumab. After treatment, the patient showed a reduction in skin damage and achieved a favorable outcome. He is still currently under follow-up observation.

## Case report

**Figure 1:**
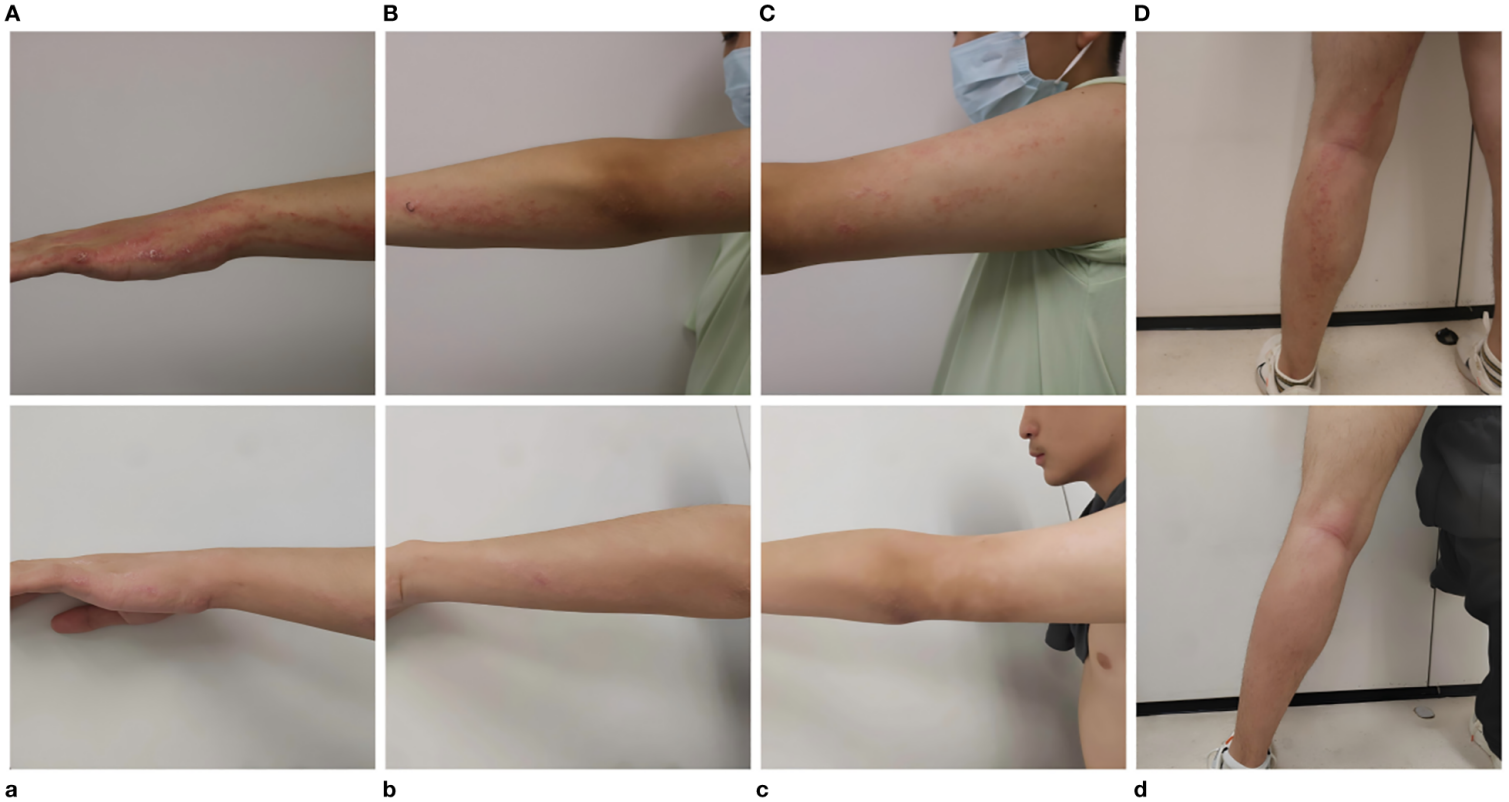
Before treatment, erythema along the Blaschko line on the patient’s left upper **(A–C)** and left lower limbs **(D)**; after two doses of ustekinumab, erythema of the left upper **(a–c)** and left lower limbs **(d)** reduced.

**Figure 2:**
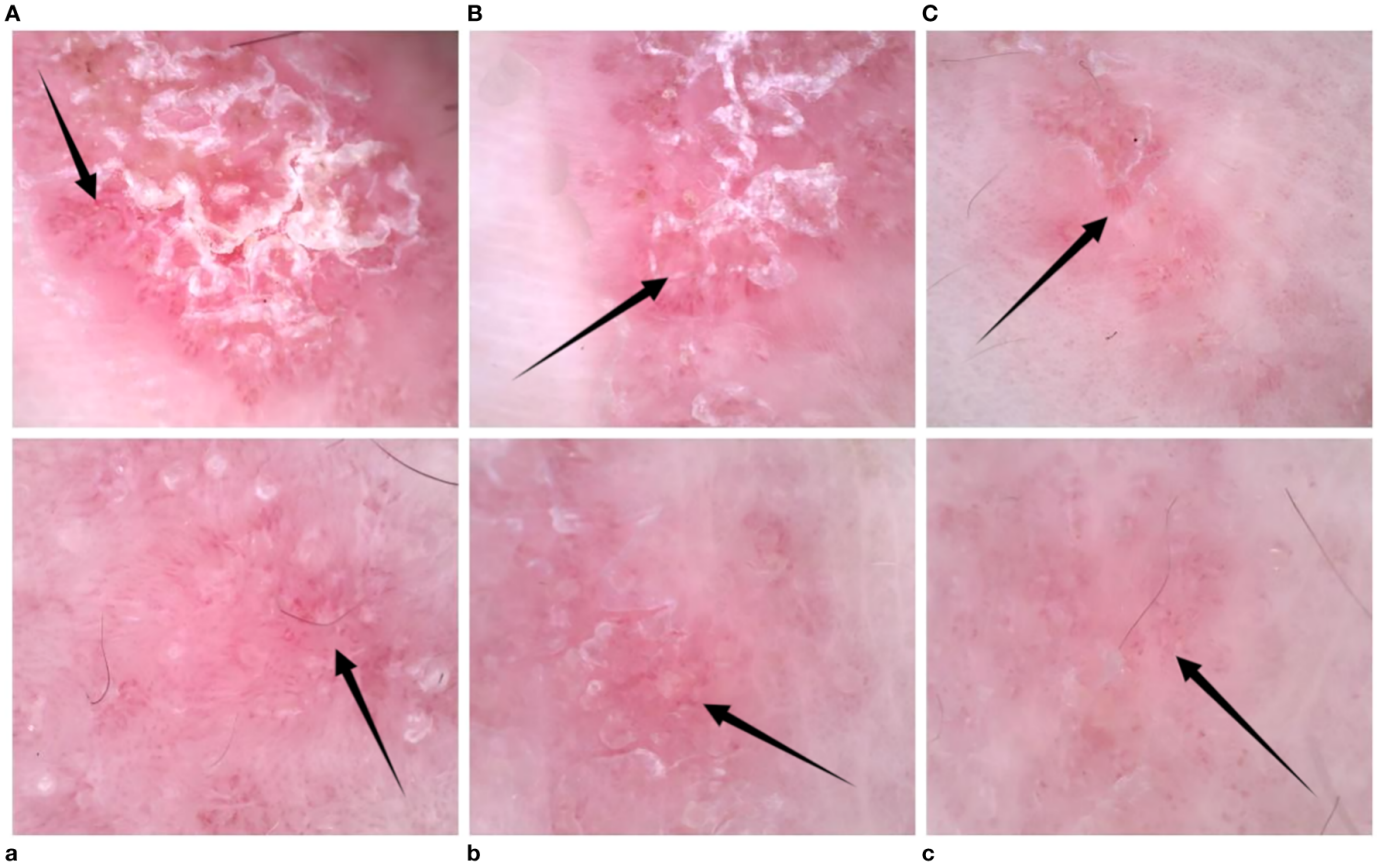
**(A–C)** Left-hand dermoscopy revealed linear erythema with reflectively arranged punctate, hairpin, and linear blood vessels visible against a red background and scaling distributed along the lines; **(a–c)** dermoscopy showed a decrease in the number of blood vessels after treatment.

**Figure 3:**
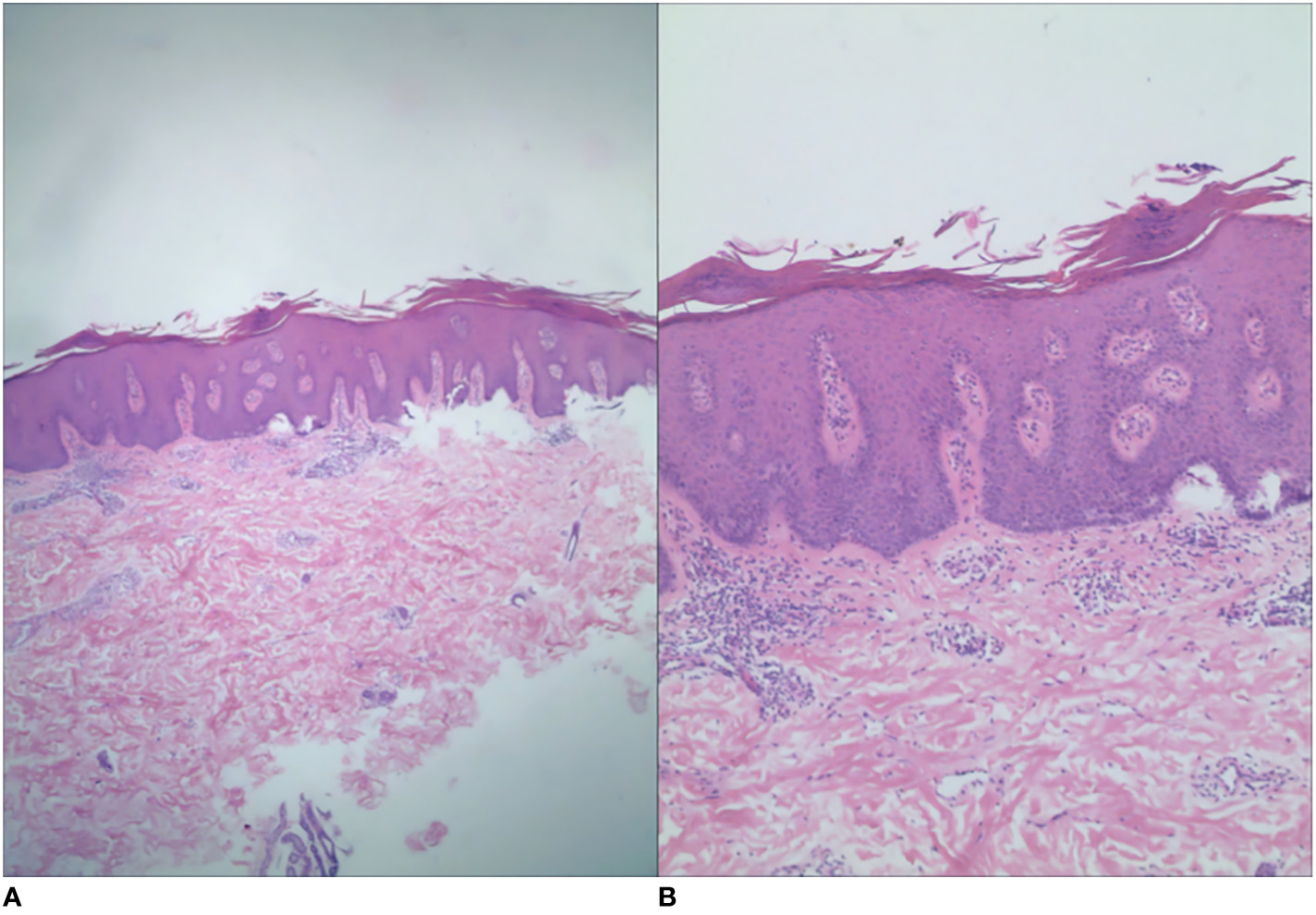
Histopathology showed hyperkeratosis and parakeratosis, shallower granular layer, Munro’s microabscesses, irregular hyperplasia of the stratum spinosum, papillary edema, and lymphocytic and histiocytic infiltration around the dermal vasculature. The histologic grading system score was 12 [**(A)** H&E, ×40; **(B)** H&E, ×100].

The patient was a 15-year-old adolescent male, measuring 1.75 m in height and weighing 75 kg. He presented with linear erythema and scaling of the left upper and left lower extremities for 1 year. The erythema first appeared on his left hand 1 year ago and then gradually spread to his left upper extremity, and similar symptoms with pruritus began to appear on his left lower extremity. During this time, he had used topical corticosteroids, tacrolimus, calcipotriol, and oral herbs, but none of them were effective. Finally, the patient came to our department. The patient had no previous underlying diseases, no history of arthralgia or related infections before the onset of the disease, and no personal or family history of psoriasis or other skin diseases. Dermatological examination revealed linearly distributed erythema and scaling on the patient’s left upper and left lower extremities (**Figure 1**). Dermoscopy revealed linear erythema with reflectively arranged punctate and linear blood vessels visible against a red background and scaling distributed along the lines (**Figure 2**). Laboratory tests and imaging examinations were conducted, including blood and urine tests, liver and kidney function tests, serological tests for hepatitis B and hepatitis C antibodies, interferon-gamma release assay (IGRA), and chest computed tomography (CT), and the results showed no abnormalities. Subsequently, the patient underwent histopathological examination of the left upper extremity (**Figure 3**). The patient was finally diagnosed with linear psoriasis.

**Figure 4:**
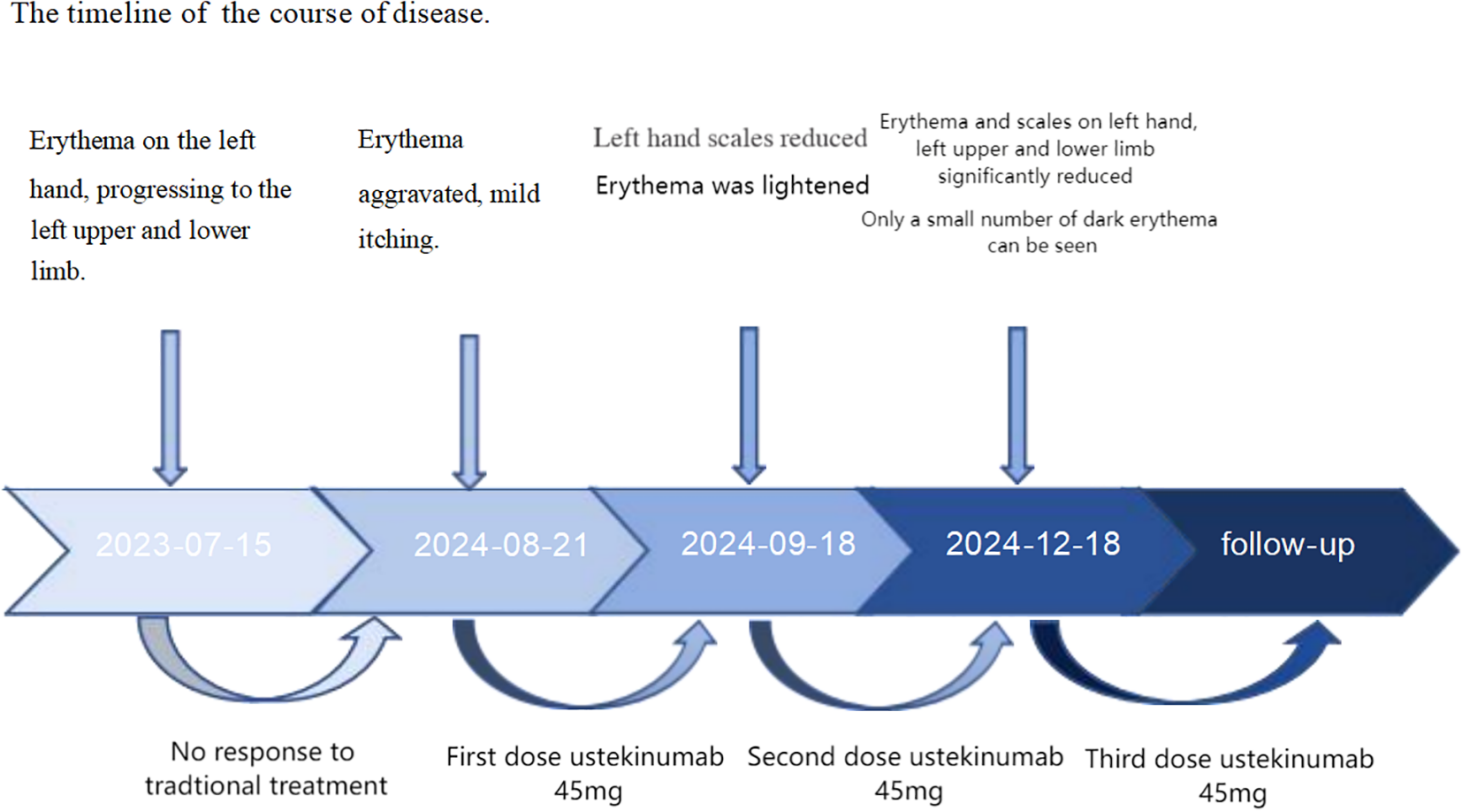
The timeline of the disease course.

Given the poor response of linear psoriasis to conventional therapies and the patient’s urgent need for effective treatment, we decided to treat him with ustekinumab. The patient received the first dose of 45 mg; 1 month later, the patient received a second dose of ustekinumab; 3 months later, the patient showed a reduction in erythema and scaling on the left upper and lower extremities (**Figure 1**), and dermoscopy showed a decrease in the number of blood vessels (**Figure 2**). His PASI and DLQI scores dropped from 3.2 to 1.8 and from 11 to 4, respectively. He was pleased with the results and agreed to receive a third dose of ustekinumab. He also became more willing to socialize and appear in public without anxiety about his appearance. We planned to refine the patient’s laboratory tests at each subsequent follow-up observation, as well as CT every 6 months, to monitor for potential side effects. Furthermore, monthly phone calls were made to ask the patient if his lesions had reduced or recurred. We sorted out the whole process according to the timeline (**Figure 4**).

## Discussion

Psoriasis is an immune-mediated, chronic, recurrent, inflammatory disease associated with genetic and environmental factors. It is typically manifested as scaly erythematous plaques or plaques, limited or widely distributed; is characterized by a wax spot sign, thin membrane, and Auspitz’s sign; and can severely affect the patient’s psychological wellbeing and quality of life, affecting approximately 2%–4% of the general population (10). Linear psoriasis is a special type of psoriasis in which the lesions often appear to be distributed in bands along the Blaschko line, which is clinically rare (1). The pathogenesis of linear psoriasis can be explained by the genetic mosaicism theory, i.e., the development of linear psoriasis lesions is associated with heterozygous deletion of its causative genes caused by mutations in early embryonic development (2). Happle also proposed the somatic cell recombination theory, which states that individuals with linear psoriasis have similar heterozygosity for multiple susceptibility genes for common psoriasis. During early embryonic development, somatic cell recombination may lead to the exchange of pathogenic gene fragments. This exchange of gene fragments may occur in cells at different stages of life. In this way, a particular somatic cell is very likely to become a homozygote for the psoriasis gene and will proliferate along the Blaschko line as a clonal stem cell, leading to the formation of localized and linearly arranged plaques of psoriasis. In combination with other susceptibility genes and environmental factors, it may eventually develop into linear psoriasis (11), and this also explains why linear psoriasis is not usually present at birth but occurs as the individual grows. There also have been isolated reports of skin lesions found that travel along the sciatic nerve, which may be related to the production of TNF-α, neuropeptides, and nerve growth factors due to local nerve root compression (12). Gene sequencing for psoriasis revealed the key role of IL-12B, IL-23R, and CARD14 genes (13, 14), and microbiome sequencing suggested that *Streptococcus* species correlate with disease severity (15). They may also be the possible mechanism of linear psoriasis.

In the study by Chen et al. (1), approximately 30% of patients with linear psoriasis reported exogenous triggers or aggravating factors, including medications, changes in climate, and infections, indicating that the occurrence of skin lesions often requires the presence of external or environmental factors. MacIntyre et al. (16) reported a case of linear psoriasis that developed after long-term use of a TNF-α inhibitor (infliximab) to treat hidradenitis suppurativa; in addition, a cohort study of over 7,000 patients showed that infliximab was associated with the highest risk of new-onset psoriasis compared with etanercept and adalimumab in the general population (17). However, it is currently not possible to determine whether using TNF-α inhibitors, especially infliximab, can induce linear psoriasis. Linear psoriasis has also been reported during the treatment of psoriasis with secukinumab (18), and a similar case has been reported in China (19). Huang et al. (3) reported a case of linear psoriasis induced by pembrolizumab in a patient with squamous lung cancer, suggesting that linear psoriasis should be considered when linear lesions develop in a patient receiving immunotherapy. Furthermore, Garg et al. (20) reported the first case of linear psoriasis induced by lithium, and this form of psoriasis was unresponsive to drugs such as topical steroids, keratolytics, and calcipotriol and may require discontinuation of lithium. Moreover, we summarized the inducements of patients with linear psoriasis among the cases reported (**Table 1**). The exact mechanism of how these factors induce linear psoriasis is still unclear because of the small number of case reports. In our case, the patient had no specific triggers, history of associated infections, relevant drug use, or personal history of psoriasis, and it was an acquired form of isolated linear psoriasis.

**Table 1:** Summary of possible causes and treatment of linear psoriasis.

Author	Age/gender	Duration of LP	Possible causes	Treatment and outcome
Huang et al. (3)	71/man	N/A	Pembrolizumab (PD-1 agent)	N/A
Ghoneim et al. (4)	25/woman	15 years	N/A	8 doses of ixekizumab Almost-complete resolution of the cutaneous lesions
Galluzzo et al. (12)	45/man	12 weeks	L5-S1 spinal nerve root compression produced TNF-α, neuropeptides, and nerve growth	Stopped running and the psoriasis spontaneously receded
MacIntyre et al. (16)	56/man	N/A	Infliximab (TNF-α inhibitor)	Changed the interval of infliximab from every 4 weeks to every 6 weeks and offered betamethasone dipropionate/calcipotriol (Enstilar) topical foam Clearing 90% of the affected areas
Saylam Kurtipek et al. (18)	33/man	N/A	Not clear, linear psoriasis occurred during the treatment of psoriasis with secukinumab (IL-17 inhibitor)	Continued to use secukinumab, topical steroids, calcipotriol, methotrexate 5 mg/week No significant improvement was observed in the lesions
Yang et al. (19)	38/man	6 months	Not clear, linear psoriasis occurred during the treatment of psoriasis with secukinumab (IL-17 inhibitor)	3 months of secukinumab, topical captopanol betamethasone ointment, and then switched to ixekizumab for 3 months The area of skin lesions was larger than before
Garg et al. (20)	36/woman	3 weeks	Lithium	Discontinued lithium and used topical clobetasol propionate and calcipotriol The lesions improved significantly over the next 2 to 3 months
Walterscheid et al. (24)	8/boy	N/A	N/A	Ixekizumab Exhibited significant clearance
Yu et al. (30)	7/boy	4 months	The mutation responsible for porokeratotic eccrine ostial and dermal duct nevus would constitute a rare but critical psoriasis gene	N/A
Baselga et al. (31)	21/woman	4 years	Linear psoriasis developed after a neurologic flare of SLE	Coal tar, dithranol, topical corticosteroids, and oral methotrexate (12.5 mg/week for 3 weeks) Linear lesion has remained essentially unchanged
Masina et al. (32)	24/woman	Few months	HIV infection	N/A
Christov et al. (33)	28/man	5 years	N/A	Calcipotriol/betamethasone 50 μg/g + 0.5 mg/g cream, calcineurin inhibitor. Followed by ixekizumab 2 × 80 mg Only residual erythema without infiltration was seen
Sun et al. (34)	42/man	3 months	Dupilumab	Adjusted dupilumab from every 2 weeks to every 4 weeks Fluticasone propionate cream and calcipotriol ointment The skin lesions gradually subsided
Martora et al. (35)	2/boy	6 months	N/A, but the patient had Down’s syndrome	Refused systemic treatment and was lost to follow-up
Klebes et al. (36)	8/boy	2 years	N/A	Topical mometasone and dithranol 1% ointments for 4 weeks A remarkable reduction in the erythema as well as the thickness of the plaques
Takahashi et al. (37)	78/man	28 years	N/A	0.12% betamethasone 17-valerate and maxacalcitol (25 μg/g) ointments for 8 weeks The lesion improved significantly but recurred 4 weeks after the cessation of the treatment
Figueiras et al. (38)	3/boy	3 months	N/A	Clobetasol propionate 0.05% for 3 weeks Significant decrease of the plaques
Brinca et al. (39)	56/woman	3 months	N/A	Betamethasone valerate and calcipotriol ointment Methotrexate 10 mg weekly and reduced to 7.5 mg weekly after 26 weeks Excellent response with no clinical relapse
Ginarte et al. (40)	25/man	N/A	Koebner phenomenon	Keratolytics and topical calcipotriol Only temporary improvement

N/A, Not applicable.

Linear psoriasis is mainly distinguished from inflammatory linear verrucous epidermal nevus (ILVEN), which typically appears at birth or develops within the first month of life, progresses relatively slowly, and is commonly seen in female patients. The typical clinical presentation of ILVEN is chronically itchy erythematous and verrucous scaly papules and plaques that are linearly distributed along the line of Blaschko, usually affecting the lower half of the body, with the buttocks being the most commonly affected area. However, ILVEN is significantly resistant to treatment, and only temporary improvement can be obtained with topical hormones, calcineurin phosphatase inhibitors, and calcipotriol; and surgical intervention may be effective for limited lesions (21). In contrast, linear psoriasis tends to start later, progresses rapidly, has less itching, and responds to conventional treatment, although not as effective as classic psoriasis (22). Ferreira et al. (23) confirmed that immunostaining for ectodermal proteins is more helpful in the differential diagnosis of ILVEN and psoriasis: ectodermal proteins are not expressed in the keratinized, underdeveloped epidermis of the epidermal nevus, but they are expressed in the basal-superficial epidermis of the psoriatic lesions. Linear psoriasis can also be differentiated from linear lichenoid dermatosis (commonly seen in children as self-manifesting linear papules), morphea linearis (without scaling, pathology shows increased collagen fiber proliferation), trauma, or contact dermatitis. When making a diagnosis, clinicians should combine the history, physical examination, laboratory tests, and treatment response to make a comprehensive analysis, and for difficult cases, long-term follow-up and dynamic evaluation are the keys to ensuring a correct diagnosis. In our case, the diagnosis of linear psoriasis can still be made based on his medical history and clinical presentation.

Currently, the treatment of linear psoriasis is challenging, and there are no guidelines or consensus for this disease. Ghoneim et al. (4) reported a 25-year-old female patient treated with ixekizumab after failure of conventional treatment regimens, and the lesions regressed completely after 4 months with only eight doses of ixekizumab. They also summarized six reports of treatment of linear psoriasis with biologics and found that linear psoriasis was refractory to etanercept, infliximab, adalimumab, and ustekinumab and responded well to ixekizumab. However, our patient responded well to ustekinumab, and it may be related to the individual physical condition or severity of the disease. Another study by Walterscheid et al. (24) reported an 8-year-old male child who was treated with ixekizumab, resulting in significant clearance of skin lesions. Pourchot et al. (25) also successfully treated a pediatric patient with linear psoriasis of the lower limbs with ixekizumab. Considering these research results, the efficacy of ixekizumab in treating linear psoriasis is worthy of affirmation. However, after reviewing relevant literature, we found that paradoxical reactions occurred after using ixekizumab to treat psoriasis: atopic dermatitis-like rash, eczema (26), and one case report showing the development of vitiligo, which may be related to its causing an imbalance in the skin immune microenvironment (27). One study showed that ustekinumab was better tolerated and durable than ixekizumab in long-term treatment (28). Moreover, ustekinumab has a lower incidence of antidrug antibodies, which may reduce the risk of treatment failure, while ixekizumab may have higher immunogenicity issues (29). So, we finally chose ustekinumab at a dose of 45 mg (in China, for children and adolescents over 6 years of age, the initial recommended dose is 45 mg for body weight between 60 and 100 kg), and it indeed showed good therapeutic effects, which might provide a new possibility for the selection of new biologics and individualized treatment for patients with linear psoriasis.

## Conclusion

Linear psoriasis is rare and difficult to treat due to its unique pathogenesis, which undoubtedly imposes a heavy burden on patients. Our case study demonstrates the potential of ustekinumab in treating this disease. However, the main limitations of our study are that it is a case report and the follow-up period is short. Additional case series or controlled clinical trials with a larger sample size are needed in the future to confirm the efficacy of ustekinumab in treating linear psoriasis and to develop standardized treatment protocols for the disease.

## Data Availability

The raw data supporting the conclusions of this article will be made available by the authors, without undue reservation.
